# Voxel-Based Dose–Toxicity Modeling for Predicting Post-Radiotherapy Toxicity: A Critical Perspective

**DOI:** 10.3390/jcm14207248

**Published:** 2025-10-14

**Authors:** Tanuj Puri

**Affiliations:** Division of Cancer Sciences, The University of Manchester, Paterson Building, Wilmslow Road, Manchester M20 4BX, UK; tanujpuri82@gmail.com or tanuj.puri@manchester.ac.uk

**Keywords:** radiotherapy, dose–toxicity modeling, predictive modeling, dose–surface maps, dose–toxicity maps, statistics, voxel-based analysis, image-based data mining, toxicity

## Abstract

This perspective paper critically examines the emerging role of voxel-based analysis (VBA), also referred to as image-based data mining (IBDM), in dose–toxicity modeling for post-radiotherapy toxicity assessment. These techniques offer promising insights into localized organ subregions associated with toxicity, yet their current application faces substantial methodological and validation challenges. Based on prior studies and practical experience, we highlight seven key limitations: (i) lack of clinical validation for dose–toxicity models, (ii) strong dependence of results on statistical method selection (parametric vs. nonparametric), (iii) insensitivity of commonly used tests to uniform dose scaling, (iv) influence of tail selection (one- vs. two-tailed tests) on statistical power, (v) frequent misapplication of permutation testing, (vi) reliance on dose as the sole predictor while neglecting patient-, treatment-, and genomic-level covariates, and (vii) misinterpretation of voxel-wise associations as causal in the absence of appropriate causal inference frameworks. Collectively, these limitations can obscure clinically relevant dose differences, inflate false-positive or false-negative findings, obscure effect direction, introduce confounded associations, and ultimately yield inconsistent identification of high-risk subregions in organs at risk and poor reproducibility across studies. Notably, current dose-only-based univariable VBA/IBDM approaches should be regarded as hypothesis-generating rather than clinical decision-making tools, as unvalidated findings risk premature translation into clinical practice. Advancing personalized radiotherapy requires rigorous outcome validation, integration of multivariable and causal modeling strategies, and incorporation of clinical and genomic data. By moving beyond dose-only predictor models, VBA/IBDM can achieve greater biological relevance, reliability, and clinical utility, supporting more precise and individualized radiotherapy strategies.

## 1. Introduction

**Figure 1:**
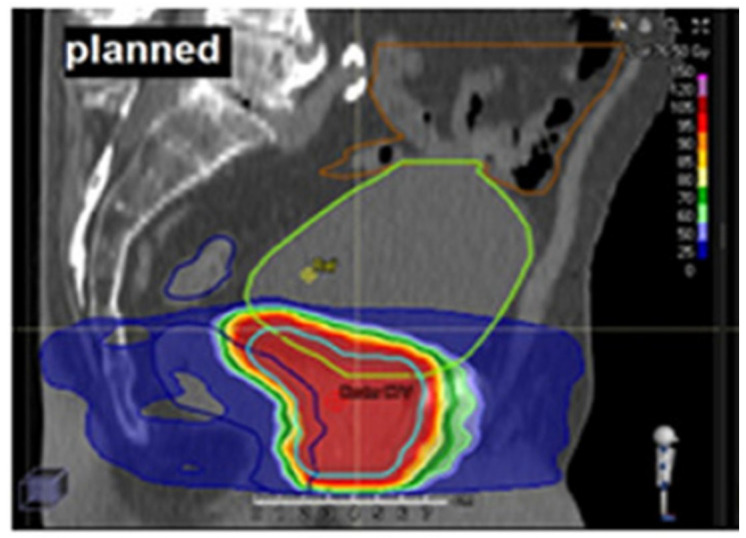
Example of a sagittal computed tomography (CT) image showing the planned radiation dose distribution to the clinical target volume (CTV), bladder, and rectum. For patients with low-risk prostate cancer, the CTV included only the prostate, whereas in intermediate-risk cases, it also encompassed 10–15 mm of the proximal seminal vesicles. A 7 mm margin was added to the CTV to define the planning target volume (PTV). The prescribed dose was 76.50 Gy, delivered in 34 fractions of 2.25 Gy each. The bladder and rectum, considered organs at risk (OARs), are contoured in light green and dark blue, respectively. Adapted from Bostel et al. [4], an open access article distributed under the Creative Commons Attribution License (CC BY).

Cancer remains a major global health burden, with radiotherapy being a cornerstone of curative treatment for many malignancies. Radiotherapy achieves its therapeutic effect primarily by inducing deoxyribonucleic acid (DNA) damage in tumor cells, ultimately leading to cell death. In external beam radiotherapy (EBRT), high-energy ionizing radiation is delivered from outside the body to a tumor inside the body. As the beam travels through the body, it inevitably also passes through and interacts with surrounding healthy tissues. To ensure that the tumor consistently receives the intended dose despite uncertainties such as internal organ motion and variations in patient positioning, the clinical target volume (CTV, which includes the tumor and a margin for microscopic disease) is expanded to form the planning target volume (PTV) [1]. While this expansion improves tumor coverage, it also increases radiation exposure to adjacent healthy tissues, known as organs at risk (OARs), as shown in Figure 1. Depending on tumor location and anatomical context, such exposure can cause radiation-induced toxicities that diminish post-treatment quality of life [2]. Therefore, improving the precision of target delineation and minimizing unnecessary dose to OARs are central aims of modern image guided radiotherapy [3].

### 1.1. Dose–Volume Histograms

**Figure 2:**
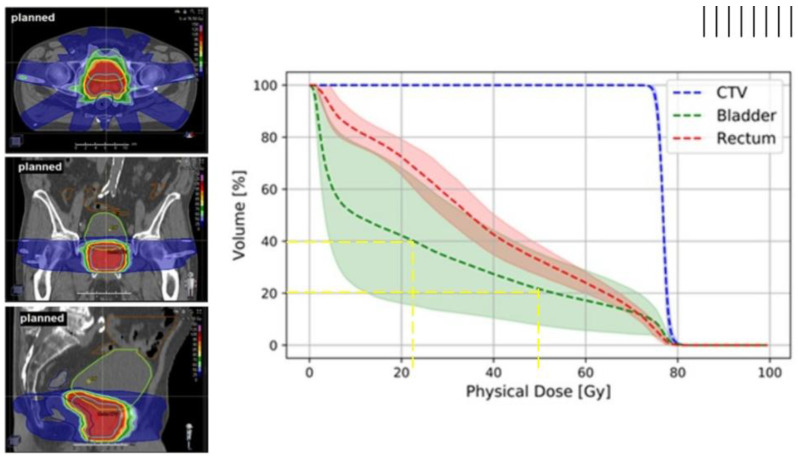
Representative transaxial, coronal, and sagittal computed tomography (CT) images (top to bottom in the left-hand panel) alongside the corresponding dose–volume histogram (DVH) from an intensity-modulated radiotherapy (IMRT) treatment plan for a prostate cancer patient. The DVH (right-hand panel) shows dose distributions for the clinical target volume (CTV, blue line), rectum (red line), and bladder (green line). Yellow dashed lines indicate that approximately 20% of the bladder volume receives ~50% of the prescribed dose (V50 ≈ 20%), and ~40% of the bladder volume receives ~22% of the prescribed dose (V22 ≈ 40%). Adapted from Bostel et al. [4]. This image is from an open access article distributed under the terms of the Creative Commons Attribution License (CC BY).

Dose–volume histograms (DVHs) are the standard tool for evaluating both PTV coverage and OAR sparing [5,6,7,8,9,10]. DVHs condense a three-dimensional (3D) dose distribution within a structure into a two-dimensional (2D) plot, showing the fraction (or absolute volume) of the organ that receives at least a given dose. DVH is constructed by calculating the dose received in every voxel of a given tissue and then summarizing this data as a cumulative plot, with dose on the x-axis and volume on the y-axis (Figure 2). Two commonly reported DVH-based metrics are Vx and Dx. The Vx metrics (e.g., V95) denote the percentage or absolute volume of a structure receiving at least x Gy, or at least x% of the prescribed dose. For example, V95 represents the proportion of the target volume covered by at least 95% of the prescribed dose. In contrast, Dx metrics describe the dose corresponding to a specified volume of the structure. When x is expressed in cubic centimeters (e.g., D2cc), it refers to the minimum dose delivered to the most irradiated 2 cm^3^ of tissue, independent of organ size. When *x* is given as a percentage (e.g., D50), it represents the minimum dose received by the most irradiated 50% of the organ. In DVH terms, this is the dose value at which 50% of the organ volume still receives at least that dose, by definition, the median dose, since half of the volume receives a higher dose and half receives a lower dose. This distinction matters: absolute-volume metrics such as D2cc quantify dose to a fixed tissue volume, facilitating comparison across organs, while relative-volume metrics such as D50 scale with organ size. Such dosimetric metrics are clinically intuitive, but they are spatially non-specific in that they do not show where within the organ the dose was delivered. Moreover, DVHs rely on simplifying assumptions that reduce biological realism, such as uniform dose distribution (ignoring regional dose variation), homogeneous tissue properties (overlooking differences in cellular sensitivity), uniform radiation response (despite known biological variability), or independence of subvolumes (ignoring interconnected organ functions and repair mechanisms). In reality, dose often varies across an organ, radiosensitivity is not uniform, and neighbouring regions can influence one another’s response [11]. Consequently, patients with similar DVHs may experience different toxicity outcomes, reflecting factors beyond dose–volume distribution that DVHs do not capture, such as genetic predisposition, comorbidities, lifestyle, or prior treatments [12,13].

### 1.2. Voxel-Based Analysis (VBA) or Image-Based Data Mining (IBDM)

VBA/IBDM, also known as voxel-based dose–toxicity analysis, was developed to address at least some of the DVH-based limitations [13,14,15,16]. Unlike DVH metrics that average dose over an entire structure or subvolume, VBA/IBDM assesses the dose at each individual voxel (the smallest unit of a 3D image) and directly associates these voxel-level doses with observed post-radiotherapy toxicity outcomes. This preserves spatial detail and enables identification of subregions within OARs that are disproportionately associated with toxicity. The approach is particularly valuable for anatomically and functionally heterogeneous OARs such as the rectum, bladder, or heart, where both dose distribution and radiosensitivity vary regionally. By aggregating voxel-wise dose–toxicity data across patients, these methods can reveal high-risk OAR subregions that might be missed by organ-level summaries such as DVHs. In practical terms, such information could be used to adapt treatment plans to spare high-risk subregions without compromising tumor control.

**Figure 3:**
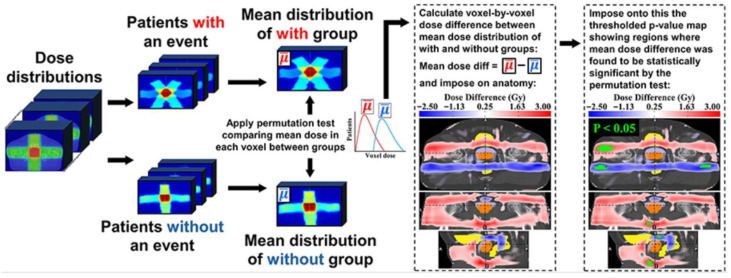
Schematic overview of the voxel-based analysis (VBA) workflow, also known as image-based data mining (IBDM), adapted from Marcello et al. [17]. The diagram illustrates the key steps involved in VBA/IBDM analysis, which form the basis of the five steps discussed in the Section 1 (Introduction). It begins with 3D dose distribution data from patients with and without a toxicity event (left), from which mean dose distributions are calculated for each group separately (center-left). A voxel-wise statistical test is first applied to generate an initial statistical map. Permutation testing is then performed to create an empirical null distribution, which is used to threshold the original statistical map, producing a thresholded *p*-value map that identifies regions where dose differences are statistically significant (*p* < 0.05, shown in green) when overlaid onto the anatomical structure (right). This voxel-based approach enables spatial identification of dose–toxicity relationships while controlling for multiple comparisons across the entire treatment volume, providing insight into which anatomical voxels may be most critical for the observed toxicity. This image is from an open access article distributed under the terms of the Creative Commons Attribution License (CC BY).

A typical VBA/IBDM workflow proceeds in five main steps (Figure 3), outlined as follows.

**Figure 4:**
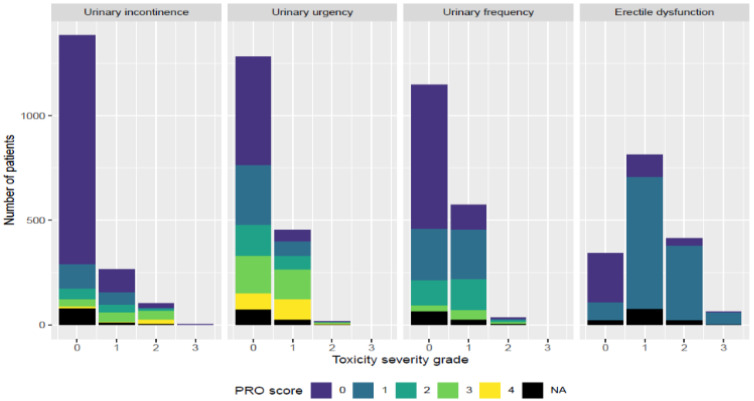
Baseline toxicities assessed by clinicians using CTCAE v4.0 grades (x-axis, varying between 0–3) are presented with overlapping patient-reported toxicities (PROs, shown in colour, varying between 0–4) from the REQUITE study (Validating pREdictive models and biomarkers of radiotherapy toxicity to reduce side effects and improve QUalITy of lifE in cancer survivors). For 1808 prostate cancer patients, genitourinary (GU) symptoms are derived from the prostate-specific PRO dataset. The image is from Seibold et al. [20], an open access article distributed under the Creative Commons Attribution—NonCommercial—NoDerivs (CC BY-NC-ND 4.0).

First, patients are typically divided into “event” (toxicity present) and “non-event” (toxicity absent) groups by dichotomizing toxicity grades (e.g., grade ≥ 1 vs. grade 0) using either baseline-corrected or uncorrected methods [18]. In the baseline-corrected approach, the maximum post-treatment grade is adjusted by subtracting the baseline grade; if ≥1, the patient is an event, otherwise non-event. This assumes (i) toxicity is attributable solely to radiotherapy, excluding pre-existing symptoms, and (ii) grades lie on a linear scale, where consecutive differences (e.g., grade 1 vs. 0 and grade 3 vs. 2) carry equal weight. Baseline correction is especially important in settings like prostate cancer genitourinary toxicity, where urinary symptoms are common pre-treatment [19] (Figure 4 shows baseline toxicity data from REQUITE trial [20]), as it prevents misattribution of baseline symptoms to radiation. In the uncorrected approach, classification relies only on the highest post-treatment grade. This combines baseline and radiation-induced symptoms, potentially inflating associations in predisposed regions. It also implicitly assumes that the toxicity scale is non-linear, with higher-grade differences (e.g., grade 3 vs. grade 2) regarded as clinically more significant than lower-grade differences (e.g., grade 2 vs. grade 1). While this approach can be more sensitive in identifying larger high-risk OAR subregions [18], it risks confounding radiation effects with unrelated factors.

**Figure 5:**
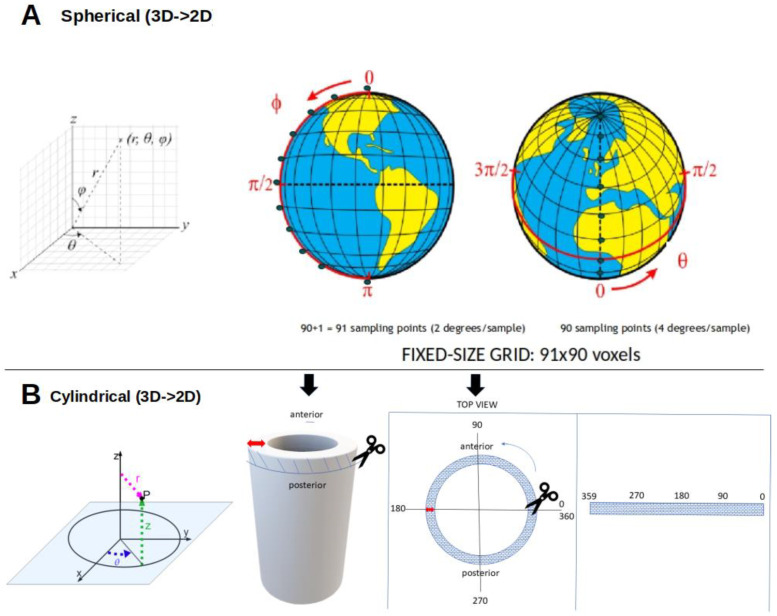
Illustration of dose–surface mapping for pelvic organs using spherical and cylindrical coordinate parameterizations (example approach as used by Puri et al. [18]; alternative methods have been reported in the literature). (**A**) Bladder mapping in spherical coordinates: The bladder surface is represented in a three-dimensional spherical coordinate system, with radial distance measured from the center of mass and angles defining orientation. The surface is modeled as a sphere and unrolled into a fixed 91 × 90 voxel grid, enabling consistent spatial normalization and direct comparison of dose–surface maps (DSMs) across patients. (**B**) Rectum mapping in cylindrical coordinates: The rectal wall is parameterized in a cylindrical system, with radius from the central axis, angle around the axis, and height along the superior–inferior direction. The surface is modeled as a cylinder and unrolled into a standardized 91 × 90 voxel grid for rectal DSM analysis.

Second, the relevant OAR is segmented from imaging (computed tomography, CT, or magnetic resonance imaging, MRI), and surface dose is mapped to a 2D dose–surface map (DSM) for hollow organs such as the bladder or rectum using cylindrical or spherical coordinates (Figure 5). To enable voxel-wise comparisons across patients, DSMs are spatially normalized either by registering images to a standard template or by dividing each DSM into identical voxel grid size (e.g., all 91 rows × 90 columns) [18,21], such that a given voxel corresponds to roughly the same anatomical location across patients.

Any mapping from a curved 3D surface to a flat 2D grid inevitably introduces geometric distortion. In spherical mapping, distortion is minimal near the model “equator” and increases toward the poles, whereas in cylindrical mapping it arises in regions of high curvature or where the surface departs from a cylindrical form. Neither method is artifact-free: spherical mapping may stretch areas depending on angular sampling, while cylindrical mapping may compress or elongate others depending on the unwrapping procedure. Such effects can subtly alter the apparent spatial distribution of dose, influencing voxel-wise statistical comparisons. Cartographic equal-area projections (e.g., Lambert cylindrical and Mollweide [22]) preserve surface area but introduce substantial shape distortion and are difficult to adapt to irregular, patient-specific organs such as the bladder. Since no equal-area projection preserves both area and shape globally, organ-tailored algorithms would be required. In practice, fixed-step spherical or cylindrical unwrapping is usually preferred despite some distortion, as it ensures reproducible point-to-point correspondence across patients and enables consistent voxel-wise analysis.

Third, dose values are often converted to biologically equivalent dose in 2 Gy fractions (EQD2) to account for fractionation and tissue-specific radiosensitivity [23,24,25]. The 2 Gy fraction is the conventional reference, adopted historically in most clinical trials, supported by dose and toxicity data, and considered to balance tumor control with normal-tissue repair. EQD2 is calculated using the linear–quadratic (LQ) model, EQD2 = D × ((d + α/β)/(2 + α/β)), where D is the total dose, d = D/n is the dose per fraction, and α/β is the dose at which the linear (α) and quadratic (β) components of cell killing are equal. The α/β ratio reflects tissue sensitivity: late-responding tissues such as the bladder and rectum have lower α/β values and are therefore more sensitive to fractionation, whereas early-responding tissues have higher α/β values and show less sensitivity. Reported α/β values vary by tissue type, toxicity, and treatment modality, underscoring the need for context-specific selection [25,26,27,28,29]. For example, in prostate cancer, α/β values of 1 Gy and 3 Gy are often used for late bladder- and rectum-related toxicities, respectively, enabling standardized voxel-wise comparisons across different fractionation schedules.

**Figure 6:**
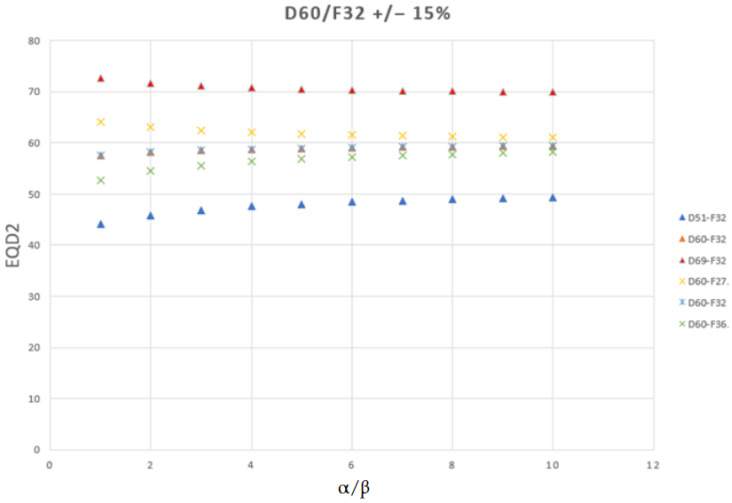
Effect of α/β ratio on Equivalent Dose in 2 Gy Fractions (EQD2) calculations (x-axis: α/β ratio in Gy; y-axis: EQD2 in Gy). The plot illustrates EQD2 values for a fixed total dose of 60 Gy delivered in 27 fractions (yellow crosses, 2.22 Gy/fraction), 32 fractions (blue crosses, 1.88 Gy/fraction), and 36 fractions (green crosses, 1.67 Gy/fraction), and also shows how three different total doses, 51 Gy (blue triangles), 60 Gy (orange triangles), and 69 Gy (red triangles), each delivered in 32 fractions, change across the same α/β range (1 to 10 Gy). The figure demonstrates how EQD2 normalization can systematically distort dose voxel values depending on both fraction size and the chosen α/β ratio.

Converting a physical dose to EQD2 using the LQ model is not a simple scaling; when the fraction size differs from 2 Gy, the equivalent dose depends jointly on the actual fraction size and the chosen α/β ratio. When fraction size exceeds 2 Gy, tissues with lower α/β ratios exhibit increased EQD2 sensitivity to fractionation, amplifying differences between regimens, whereas tissues with higher α/β ratios show reduced sensitivity, resulting in smaller variations (Figure 6). For instance, a schedule of 30 × 2 Gy always corresponds to 60 Gy EQD2, regardless of the chosen α/β ratio. This α/β independence occurs because at 2 Gy per fraction, the EQD2 formula reduces to a direct equality with the total physical dose. In contrast, fractionation schemes with doses per fraction different from 2 Gy show α/β dependence. For example, 20 × 3 Gy equals about 72 Gy EQD2 when α/β = 3 Gy (typical for late rectum toxicity) and about 80 Gy when α/β = 1 Gy (typical for later bladder toxicity). The same principle applies to a total dose of 60 Gy delivered in different fraction numbers. With 27 fractions (2.22 Gy per fraction), the EQD2 decreases as α/β increases, from 64.4 Gy at α/β = 1 Gy to 61.1 Gy at α/β = 10 Gy. Conversely, when 60 Gy is delivered in 32 fractions (1.88 Gy per fraction) or 36 fractions (1.67 Gy per fraction), the EQD2 increases as α/β increases. For 32 fractions, EQD2 rises from 57.8 Gy at α/β = 1 Gy to 59.6 Gy at α/β = 10 Gy. For 36 fractions, it rises from 53.5 Gy at α/β = 1 Gy to 58.5 Gy at α/β = 10 Gy.

These shifts not only magnify or compress apparent biological dose differences between event and nonevent groups but also affect voxel-wise statistics: under identical conditions, the spatial extent of significant high-risk voxels identified with VBA/IBDM depends on both fraction size and α/β (except at a fraction size of 2 Gy). For fraction doses > 2 Gy, lower α/β increases EQD2 and enlarges high-risk OAR subregions due to greater dose variation; for doses < 2 Gy, the opposite occurs. As a result, OAR subregions deemed high-risk at one α/β may differ at another, even when the physical dose is identical. Therefore, consistent, endpoint-specific α/β selection and transparent reporting of both the applied transformation and absolute dose ranges are essential for reliable analysis and interpretation.

Fourth, statistical tests are applied independently at each voxel to compare event and non-event groups. Normality of raw voxel dose values in each group is often assessed with the Shapiro–Wilk test [30]. If both groups plausibly satisfy normality and sample sizes are adequate, a parametric test (such as Student’s t or Welch’s t) [31] is used; otherwise a nonparametric test (such as Mann–Whitney U) [32] is applied. For Welch’s *t*-test, the null hypothesis is equal group means (μ_1_ = μ_2_), with the statistic t = (X_1_ − X_2_)/√((S_1_^2^/N_1_) + (S_2_^2^/N_2_)), where X_1_, X_2_ are group means, S_1_^2^, S_2_^2^ are sample variances, and N_1_, N_2_ are group sizes. The Mann–Whitney U test instead assumes the null hypothesis that the probability of a randomly selected value from one group exceeding a value from the other is 0.5. It ranks all values, sums ranks per group, and computes U_1_ = N_1_ × N_2_ + N_1_(N_1_ + 1)/2 − R_1_, with R_1_ the rank sum for group 1 (U_2_ analogously), using the smaller U for inference. These voxel-wise tests generate statistical maps (of the same voxel grid size as the original standardized template) of t- or U-values.

**Figure 7:**
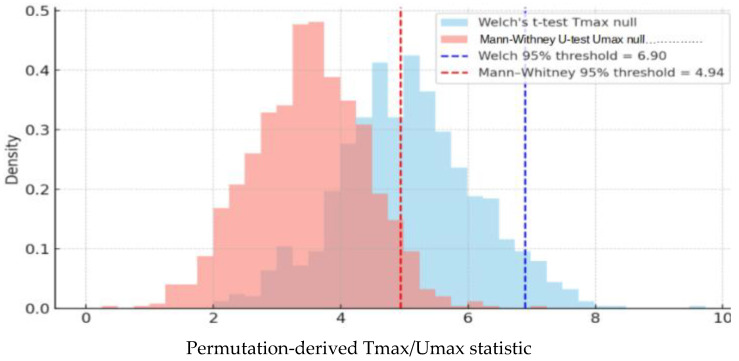
A simulated example of permutation-derived null distributions of the maximum voxel-wise test statistic for Welch’s *t*-test (Tmax) and the Mann–Whitney U test (Umax). Each histogram shows the empirical distribution of Tmax/Umax values obtained from repeated random permutations of group labels, representing the null hypothesis of no association between dose and toxicity anywhere in the map. The x-axis shows the maximum statistic observed across all voxels in each permutation, and the y-axis shows the relative frequency (density), i.e., the proportion of occurrences in each bin relative to the total number of observations. The density is scaled so that the total area under the curve equals 1, indicating how common those maximum values are in relative terms. Smooth curves overlay the histograms to aid visual comparison between tests. Dashed vertical lines indicate the 95th percentile thresholds for each distribution, which are used to control the family-wise error rate (FWER, i.e., probability of making at least one false positive) at α = 0.05 in voxel-based analyses. These thresholds are typically applied to the real (unpermuted) statistical map so that the probability of incorrectly declaring at least one voxel significant under the null remains ≤5%. Differences in distribution width and tail heaviness reflect how each statistical model responds to the same data structure, which can affect sensitivity and robustness in spatial inference.

Fifth, to control false positives, VBA/IBDM studies typically use permutation-based multiple-comparison correction. Spatial correlation between voxels is accounted for when permutation is combined with other spatially informed methods [33,34,35,36,37]. Group labels (e.g., event vs. non-event) are repeatedly shuffled to generate datasets without true dose–toxicity associations. For each permutation, voxel-wise statistics (t-values or U-values) are computed, and only the maximum value across voxels (Tmax or Umax) is retained, forming an empirical null distribution [35,38,39] against which the observed test statistic from the real (unshuffled) data is then compared; a voxel is considered significant if its statistic exceeds the chosen percentile threshold (e.g., 95th) of the null distribution used for statistical thresholding (Figure 7).

The result of this five-step process is a voxel-wise statistical map identifying anatomical subregions where local dose is significantly associated with toxicity. Such maps have been used to study both dose–survival [40,41] and dose–toxicity relationships [14,15,42]. This paper focuses on dose–toxicity (rather than dose–survival) modeling, with the goal of generating dose–toxicity maps that, once validated, could guide personalized planning to spare high-risk OAR subregions and ultimately improve patient toxicity outcomes.

The aim of this perspective paper is to critically examine the current methodological challenges and limitations of VBA/IBDM. We highlight some common pitfalls in statistical analysis, interpretation, and validation, such as those illustrated in this paper, and offer practical guidance for researchers applying these techniques. This work emphasizes that univariable VBA/IBDM approaches should be regarded as hypothesis-generating tools rather than definitive instruments for clinical decision-making. Moreover, we underscore the importance of rigorous outcome validation, incorporation of multivariable and causal modeling strategies, and integration of clinical and genomic data, identifying key directions for future research to enhance the biological relevance, reliability, and clinical utility of such VBA/IBDM studies in radiotherapy.

## 2. Problem

VBA/IBDM evaluates voxel-level dose distributions in relation to toxicity, preserving anatomical detail and identifying OAR subregions most strongly associated with adverse toxicity effects. Unlike DVHs, which reduce complex dose information to summary statistics, these methods capture spatial dose–toxicity relationships, potentially guiding strategies to spare high-risk OAR subregions without compromising tumor control. Therefore, this manuscript concentrates on seven technically intense methodological challenges of particular importance based on our prior experience.

(i) Lack of clinical validation of dose–toxicity models. This remains the most critical barrier because, without outcome-based validation demonstrating clinical benefit, statistical maps are hypothesis-generating only and cannot be directly translated into practice.
(ii) Choice between parametric and non-parametric models, because model choice influences sensitivity to identify high-risk subregions within OARs, and robustness to assumption violations, thereby affecting reproducibility.
(iii) Sensitivity of statistical models to uniform dose scaling, which can obscure clinically relevant differences when the dose scale changes.
(iv) Choice of one-tailed versus two-tailed tests, which affects statistical power, false-positive rates, and interpretability.
(v) Correct implementation of permutation testing to control the family-wise error rate (FWER). Improper use can inflate false positives or reduce interpretability.
(vi) Use of dose-only predictors, which ignores patient, treatment, genomic, anatomical, and other clinical covariates that may confound dose–toxicity associations.
(vii) Interpreting associations as causal without an appropriate framework, which risks misattribution of toxicity to dose when associations may instead be driven by confounding, bias, or artefacts.

These challenges were prioritized because they directly affect robustness, reproducibility, and clinical relevance. Other limitations, such as site-specific artefacts or other technical issues, may have less generalizable impact. Each of these challenges is discussed in detail in the subsequent Section 3.

## 3. Discussion

### 3.1. Dose–Toxicity Modeling Lacking Clinical Validation

VBA/IBDM are increasingly applied to identify high-risk subregions within OARs where radiation dose is associated with post-treatment toxicity. These methods are attractive because they retain spatial dose information discarded by conventional DVHs. Despite their expanding use in research across cancer types and anatomical sites, their clinical role remains uncertain due to lack of clinical validation.

True clinical validation requires going beyond retrospective reproducibility, but it should not demand prospective randomized trials as the sole standard. The appropriate benchmark is whether incorporating high-risk subregions into treatment planning (e.g., via optimization priorities or dose constraints) leads to measurable improvements in patient toxicity severity. Such evidence may come from randomized trials, but also from carefully designed pragmatic, comparative, or multi-institutional studies. Definitive proof of strict biological causality is not always necessary; in many cases, demonstrating clinical utility, showing that an intervention measurably benefits patients, may be a more appropriate threshold for clinical translation than requiring complete mechanistic certainty (i.e., a full understanding of the precise biological pathways by which the intervention produces its effect).

Clinical integration is best viewed as a continuum rather than a rigid categorization. At one end, exploratory use (e.g., hypothesis generation analyses or association modeling) is appropriate. Moving along the spectrum, informative use (e.g., guiding which planning objectives are prioritized) may be reasonable in selected contexts, provided limitations are acknowledged. At the highest level, directive use (e.g., enforcing VBA-derived constraints in treatment planning) demands a higher evidentiary bar, since systematic bias or artefacts could directly harm patients.

Despite promising advances, several limitations constrain the generalizability of dose–toxicity modeling. Model performance and reproducibility depend on data quality and methodological choices, such as patient selection, imaging protocols, segmentation, dose–surface mapping algorithm, voxel size, interpolation methods, dose calculation, image registration, and statistical analysis. Voxel-averaged dose values also assume uniform distribution and tissue homogeneity within each voxel, reducing biological plausibility. In prostate cancer studies of rectal OARs, for instance, variation in deformation models, dose metrics (planned vs. delivered), and analytic methods has been shown to produce divergent voxel-level dose–toxicity associations [43,44]. More broadly, variability across studies, compounded by the absence of quantitative criteria for defining high-risk subregions, prevents meaningful cross-study comparison and thus limits generalizability [45].

Progress toward clinical validation is further undermined by selective reporting and publication bias: negative findings are rarely published, and small, interconnected research groups repeatedly test similar pipelines on limited datasets. This fosters methodological circularity, where reproducibility reflects recycled assumptions rather than robust conclusions [46,47,48,49,50,51,52]. Rerunning similar pipelines on independent institutional cohorts and finding concordant maps demonstrates reproducibility under shared methodological assumptions, not validation. Such assessments do not establish whether the implicated regions are biologically meaningful or clinically actionable. Apparent agreement may simply reflect common artefacts in the radiotherapy pathway, such as image registration [21,53,54,55], organ segmentation [56,57,58,59], dose calculation errors [60,61,62,63] or statistical biases shared across groups, rather than genuine biological associations. Clinical validation would require prospective demonstration that sparing these regions reduces toxicity. Thus, cross-dataset reproducibility, while informative, should not be misinterpreted as validation. Such pseudo-reproducibility risks creating a false sense of robustness and could prematurely encourage clinical translation. Important patient-specific factors, such as age, irradiated OAR volume, genomic variation, comorbidities, prior surgery, and lifestyle, are often not incorporated in prediction models, despite strong evidence of their influence on toxicity risk [53,54,55,64,65,66,67,68,69,70,71,72,73,74,75,76,77,78,79,80,81,82,83,84,85,86,87,88,89,90,91]. Genetic variation may shift high-risk subregions from lower- to higher-dose regions, suggesting that models integrating genomics with dosimetry could capture mechanisms missed by dose-only approaches [92,93]. These limitations collectively explain why, despite technical advances, dose–toxicity modeling still lacks robust clinical validation and cannot yet be reliably implemented in routine practice.

Because no standardized frameworks or reference standards exist, it remains uncertain which methods are reliable, generalizable, or even merit validation. This gap underscores the importance of developing standardized integrative approaches that extend beyond dose-only models. In practice, this highlights the need for harmonized data collection, preprocessing, and analysis pipelines capable of integrating dose, genomic, and other datasets as predictors of toxicity. For VBA/IBDM to mature from exploratory tools into credible clinical decision aids, the field must first address key methodological flaws and achieve consensus on various technical and validation standards. Only with this foundation, and with outcome-driven studies showing that minimizing dose to VBA/IBDM-identified high-risk subregions meaningfully reduces post-radiotherapy toxicities, should directive use in treatment planning be considered. Until then, the appropriate role of dose–toxicity mapping remains primarily exploratory or hypothesis-generating.

### 3.2. Choosing Between Parametric and Nonparametric Statistical Models

In VBA/IBDM, the choice between parametric and non-parametric statistical models is critical, because it determines how dose–toxicity associations are tested and how robust results are to assumption violations. Parametric tests such as Student’s and Welch’s *t*-tests assume normally distributed values within each group. Student’s *t*-test additionally requires equal variances, whereas Welch’s test relaxes this assumption, remaining reliable with unequal variances or unbalanced sample sizes. Under the null hypothesis, and if assumptions hold, the test statistic *t* (not the raw data) follows a t-distribution, i.e., the sampling distribution obtained if independent samples were repeatedly drawn and the statistic recalculated.

Raw voxel dose values are often skewed or heavy-tailed. The Central Limit Theorem (CLT) states that, when voxel doses vary independently across patients and have finite variance, the distribution of the sample mean converges toward approximately normality as group size increases. Whether CLT assumptions are sufficiently met in practice directly influences the suitability of parametric tests, as violation of these conditions may necessitate non-parametric alternatives. This convergence to normality may require very large sample sizes, and highly asymmetric dose distributions (high skewness), heavy-tailed distributions with extreme values (high kurtosis), or large imbalances in the number of patients with versus without toxicity may undermine the approximation [94,95,96]. Rules of thumb such as “*n* ≈ 30” are inadequate. Because population variance is typically unknown in dose–toxicity analyses, inference relies on the t-distribution rather than the normal distribution, and much larger per-group sizes may be required for valid inference when dose distributions are highly skewed or heavy-tailed. Crucially, reliability depends on per-group size: a large cohort cannot compensate for a very small event group (e.g., 9 events vs. 200 non-events), which leaves the *t*-distribution approximation unstable. Overall sample size thresholds (e.g., ≥200 patients with ≥20% toxicity rate, implying ≥40 toxicity cases and ≥160 non-toxicity cases) must ensure both groups are large enough for stable voxelwise analysis to be meaningful. Below these levels, especially when the event group is <30–40 patients, VBA/IBDM studies are likely underpowered, and voxel-level inference becomes unreliable. In such cases, analyses at a coarser spatial scale may be preferable, such as averaging dose over predefined anatomical structures or larger anatomical regions. This reduces voxel-level noise and stabilizes inference when the toxicity group is too small for reliable VBA/IBDM.

Because only a single dataset per voxel is available, the sampling distribution of mean dose differences between events and nonevents cannot be directly observed. Automated normality tests such as Shapiro–Wilk have limited value: they are underpowered in small samples and unreliable when applied across thousands of voxels, where the large number of simultaneous tests inflates false positives. Instead of relying on automated cutoffs, graphical diagnostics (e.g., histograms and Q–Q plots) and global summaries may be more informative. Modern approaches such as bootstrap resampling and robust statistics (e.g., trimmed means, M-estimators) can provide more flexible ways to assess sampling variability and may help mitigate the impact of non-normality [97,98,99,100], thereby informing whether parametric tests remain appropriate or whether non-parametric alternatives are preferable.

Nonparametric models such as the Mann–Whitney U test evaluate whether distributions differ (often interpreted as differences in medians) by ranking observations and comparing rank sums between groups, rather than testing equality of means directly. Because they rely only on ranks, these methods make fewer distributional assumptions and are robust to skewness, outliers, and unequal variances, though offer less powerful conclusions when parametric assumptions hold. In VBA/IBDM, they are preferable when dose distributions are skewed or sample sizes are insufficient for parametric tests.

When applied voxelwise, a naïve approach of testing normality separately for each voxel for and then switching between parametric and nonparametric tests accordingly is rarely advisable, since noisy voxel-level diagnostics distort the null distribution and inflate false positives. More reliable strategies are: (i) choose one test type to apply consistently across all voxels, guided by sample sizes, group balance, variance structure, and distributional characteristics; or (ii) use distribution-free resampling methods such as permutation or bootstrap to generate valid voxelwise significance tests without relying on parametric assumptions [97,98,99,100].

A pragmatic approach to model selection in VBA/IBDM is to consider per-group sample sizes alongside global summaries of the raw voxel dose distributions. If groups are small or unbalanced and data at most voxel locations violate normality, nonparametric tests are generally preferable. If groups are moderate to large and reasonably balanced, parametric tests are typically robust due to the CLT. In mixed situations, parametric methods can still be appropriate, but sensitivity analyses using nonparametric, distribution-free sampling or resampling methods are recommended. Automated normality testing should be considered only one criterion in this process. Other factors include variance heterogeneity (favoring Welch’s t over Student’s t), the spatial distribution of voxels failing normality (to assess whether violations are systematic or localized), and whether the spatial pattern of significant voxels aligns with known anatomical and toxicity mechanisms, providing biological plausibility. Confidence in VBA/IBDM findings can be strengthened when both parametric and nonparametric tests identify the same high-risk regions. Divergence between methods indicates results may be fragile and highly dependent on assumptions, highlighting the complementary nature of parametric and nonparametric approaches, as strong effects are generally detected consistently across methods. Equally important is transparent reporting of whether alternative approaches, such as bootstrap, permutation, or robust statistics, yield consistent conclusions.

### 3.3. Statistical Invariance to Dose Scaling or Shift

Many voxel-wise statistical tests used in VBA/IBDM are invariant to uniform scaling or shifting of dose values, for both parametric tests and nonparametric tests [31,101,102,103]. While mathematically convenient, this property can be misleading because radiation toxicity risk depends on absolute dose, not merely relative group differences.

#### 3.3.1. Scale and Shift Invariance in Common Tests

Welch’s *t*-test evaluates the difference between two group means relative to within-group variability. Under scale invariance, multiplying all doses by a positive constant leaves the t-statistic unchanged, since both the numerator (mean difference) and denominator (standard error) scale proportionally. In a two-tailed test, the *p*-value depends only on the absolute value of the statistic and thus remains unchanged. In a one-tailed test, the *p*-value also remains unchanged because positive scaling preserves the direction of the mean difference. Under shift invariance, adding the same constant to both groups raises their means equally without altering the difference or variance, so the t-statistic and *p*-value remain unchanged.

The Mann–Whitney U test depends only on the rank ordering of values. Multiplying all doses by a positive constant or adding the same constant preserves ranks, so the U statistic and *p*-value remain unchanged. More generally, the test is invariant to any strictly monotonic transformation, such as logarithm, exponential, or power [101]. In two-tailed tests, the *p*-value depends only on whether the distributions differ, not the direction, and remains unaffected. In one-tailed tests, the sign of the difference determines which group is considered higher; positive scaling preserves this order, so conclusions remain consistent.

For example, if event and non-event groups have mean doses of 50 Gy and 52 Gy, doubling values to 100 Gy and 104 Gy preserves the test results, while adding 5 Gy (55 Gy vs. 57 Gy) also yields the same outcome. In practice, negative scaling would reverse the direction and change one-tailed conclusions, it is irrelevant for radiation dose, so invariance under positive transformations is sufficient to describe these tests.

#### 3.3.2. Clinical Pitfalls of Scale Invariance

These properties create clinical pitfalls. First, they can cause false equivalence across cohorts: two institutions may produce similar voxel-wise significance maps even if one prescribes higher absolute doses (e.g., 60–66 Gy vs. 74–80 Gy). Relative differences drive the statistics, but absolute risk is higher in the latter cohort. In extreme cases, a 30 Gy exposure could yield the same significance as a 60 Gy exposure, despite very different clinical implications. Second, scale invariance can lead to low-dose artefacts: a region may be flagged significant because event patients consistently received slightly higher doses than non-event patients, even if those doses are far below plausible toxicity thresholds.

#### 3.3.3. Mitigating Invariance at the Model Level

Standard voxel-wise (Welch t or Mann–Whitney U) tests also ignore nonlinear dose–toxicity relationships, volume effects, and patient-level modifiers such as baseline function or genetics, etc., potentially masking true patterns. Addressing these limitations requires modifying the statistical model rather than merely reporting Gy values or masking implausible regions. Possible strategies include voxel-wise logistic regression or Cox models with continuous dose predictors (linear or spline-transformed), inclusion of prescription-level covariates to anchor between-cohort differences, or hierarchical models with cohort-specific intercepts/slopes [104,105]. These methods could explicitly test dose–toxicity associations in Gy, reducing paradoxical equivalence across dose ranges. Model-level remedies are mainly needed when EQD2 conversion is unreliable.

#### 3.3.4. When Scale Invariance Is Appropriate: The EQD2 Context

Scale invariance is problematic for raw physical doses but can be biologically meaningful after valid EQD2 normalization, provided the normalization produces overlapping biological dose ranges across cohorts and plausible dose levels for the toxicity endpoint [18,45]. The key is ensuring normalization assumptions (e.g., justified α/β values) are valid and clearly reported. Analyses should report absolute EQD2 distributions in significant high-risk voxels, as differences at implausibly low doses may lack clinical relevance. Interpretation should focus on whether implicated subregions represent consistent anatomical vulnerabilities across regimens and whether absolute EQD2 levels are biologically plausible for the endpoint. Direct cohort-to-cohort comparisons assess whether dose–toxicity relationships and absolute EQD2 differences are consistent between centers. Pooled analyses combine data from multiple centers into a single voxel-wise model to identify high-risk subregions consistently associated with toxicity across cohorts. Both require sufficient overlap of EQD2 distributions to ensure observed associations reflect comparable biological exposures rather than center-specific protocols. When overlap is limited, trimming patients outside the common support range and accounting for cohort effects through stratification or model-based adjustments can help ensure observed differences reflect biological effects rather than protocol variation. If common support is lacking or results shift substantially with α/β variation, findings should be considered hypothesis-generating only. Without these safeguards, EQD2 anchoring or stratified analyses are more biologically defensible approaches.

In this context, anchoring refers to fixing calculations or treatment decisions to a common reference point as a framework for comparing radiotherapy regimens. It involves defining a standard EQD2 schedule as a reference and evaluating alternative fractionation schemes relative to this benchmark. Because EQD2 depends on the α/β ratio, anchoring must be applied separately for each tissue, with the tumor prescription anchored to its specific α/β and OARs evaluated against their own α/β-based tolerance limits. Multi-tissue scenarios can create inherent challenges. OARs often lie immediately adjacent to or partially within the planning target volume, resulting in competing optimization objectives that cannot always be satisfied simultaneously. Furthermore, OARs may exhibit both early and late toxicity endpoints, which correspond to different α/β ratios, early effects typically requiring higher α/β values (≈8–10 Gy) and late effects lower values (≈1–3 Gy). This introduces multiple, sometimes conflicting, reference points for the same anatomical structures, making EQD2 anchoring a multi-dimensional optimization problem that cannot be fully resolved by reference points alone. This approach builds on the well-established LQ model and standard use of EQD2 for comparing fractionation schedules and OAR constraints [23,24,25] but extends it by treating EQD2 as a fixed benchmark per tissue type rather than only as a relative comparator. In the absence of additional safety adjustments, absolute EQD2 anchoring provides a biologically consistent and transparent method for assessing tumor prescriptions and normal tissue sparing, though clinical judgment is required to balance competing endpoints.

For example, in prostate radiotherapy, a conventional schedule of 60 Gy in 30 fractions (2 Gy per fraction) can serve as the reference. Using an α/β of 1.5 Gy for the prostate tumor, this corresponds to an EQD2 of 60 Gy for the target. The same schedule corresponds to an EQD2 of 60 Gy for late-responding tissues such as the rectum and bladder (α/β = 3 Gy) and likewise for early-responding OARs with α/β ≈ 8–10 Gy. Because the fraction size is 2 Gy, the EQD2 for all tissues is equal to the physical dose regardless of α/β, making the 60 Gy in 30 fractions schedule a stable reference point, or baseline anchor, for comparison with alternative fractionation regimens. To illustrate the impact of tissue-specific α/β, consider two hypofractionated schedules. A schedule of 45 Gy in 15 fractions (3 Gy per fraction) yields an EQD2 of approximately 57.9 Gy for the prostate tumor (α/β = 1.5 Gy), 54 Gy for late-responding OARs (α/β = 3 Gy), and 48.8 Gy for early-responding OARs (α/β ≈ 10 Gy), biologically milder than the reference across all tissues. In contrast, 60 Gy in 20 fractions (3 Gy per fraction) corresponds to an EQD2 of approximately 77.1 Gy for the tumor, 72 Gy for late-responding OARs, and 65 Gy for early-responding OARs, substantially escalated despite the same total physical dose as the reference, reflecting how fraction size modulates biological dose differently across tissues with varying α/β ratios.

An additional limitation is that EQD2 anchoring focuses primarily on fraction size adjustments but does not always capture the organ-level dose–toxicity metrics most predictive of toxicity. For many OARs, risk correlates more strongly with the mean organ dose (MOD) than with maximum point doses. Recent work in lung cancer has shown that converting MODs directly to EQD2 can systematically underestimate toxicity risk, because the assumption of a universal 2 Gy anchor does not hold across different tissues. A refined approach, termed EQDd [106], uses an empirically determined “effective fraction size” (d) that aligns biological equivalent mean doses with observed toxicity outcomes. For example, an effective d of ~1.3 Gy was identified for the lung, with organ-specific relationships also reported for the esophagus and heart. These findings suggest that OAR evaluation may require organ-specific anchors rather than a uniform 2 Gy baseline, improving the biological plausibility of EQD-based toxicity assessment.

While EQD2 anchoring provides a structured framework for comparing fractionation schemes, it cannot resolve the fundamental conflicts inherent in multi-objective optimization. Tumor control and multiple OAR constraints often have incompatible requirements, and the method does not provide guidance on prioritization when anchors conflict. Moreover, it risks suggesting a level of precision that may not reflect biological reality, given variability in α/β ratios, interfractional organ motion, and patient-specific genomic sensitivity. Thus, EQD2 anchoring is best viewed as a comparative or planning tool: it can help quantify trade-offs between tumor control and toxicity and provide transparency when evaluating alternative regimens, but it should not be treated as a prescriptive optimization framework. Future refinements (such as MOD-based approaches and organ-specific effective fraction sizes) may enhance its utility, but expert judgment will remain essential for balancing competing objectives in radiotherapy planning.

### 3.4. Tail Choice in Hypothesis Testing

In VBA/IBDM, the biologically plausible hypothesis is typically directional: higher dose is expected to increase the probability of toxicity (assuming genomics and other factors remain the same). A one-tailed test reflects this expectation by assigning the entire significance level (e.g., α = 0.05) to one end of the null distribution. Here, α (alpha) is the probability of a Type I error. Concentrating α in one tail lowers the critical threshold for significance in the hypothesized direction and thereby increases the test’s power, but this comes at the cost of inability to detect effects in the opposite direction. Since power equals 1 − β, where β is the probability of failing to detect a true effect (Type II error), a one-tailed test allocates the entire α threshold to the direction of interest rather than splitting it between two tails. This increases the critical region in the hypothesized direction, reducing β and thus improving power.

This difference can be illustrated with the standard normal distribution. At α = 0.05, the critical value for a one-tailed test is *Z* = 1.645, while for a two-tailed test it is *Z* = 1.96 (because α is split into 0.025 per tail). Thus, to reject the null hypothesis in the hypothesized direction (in positive direction), the observed test statistic must be larger than 1.645 in the one-tailed case but larger than 1.96 in the two-tailed case. Since the threshold is stricter for the two-tailed test, the probability of failing to reject the null when the alternative is true (i.e., β) is higher, and therefore, power (i.e., 1 − β) is lower compared to the one-tailed test. The trade-off is that the two-tailed test can also detect effects in the opposite direction, though such “inverse” associations (e.g., lower doses appearing associated with higher toxicity) are typically implausible for late radiation effects, even if they may occasionally arise from confounding or patient-specific variability.

Although one-tailed testing can increase statistical power when a directional hypothesis is biologically well supported, this does not imply that it is always appropriate. Radiation responses are not invariably monotonic, and several biological and clinical considerations argue for retaining two-tailed flexibility. At low doses, tissues may exhibit protective or adaptive responses, where modest exposure stimulates repair mechanisms that reduce damage. This phenomenon, known as hormesis, has been reported in experimental studies and can make low-dose exposure appear less harmful than no exposure at all [107,108,109]. Clinical confounding can also complicate interpretation. For example, patients with pre-existing deficits are often prescribed lower doses for safety reasons, yet remain at higher risk of toxicity due to their underlying vulnerability. In such cases, a naïve analysis may misleadingly suggest that lower dose is associated with higher toxicity risk. This is a form of confounding by indication, where the clinical rationale for assigning a lower dose (the patient’s frailty) is itself associated with the outcome (toxicity), thereby biasing the apparent dose–toxicity relationship.

For these reasons, one-tailed testing is often justified in radiation toxicity studies but should not be regarded as universally appropriate. Investigators should pre-specify their choice of tail based on mechanistic plausibility and study aims and explicitly justify any departures from the default directional assumption. In exploratory settings, or when protective or compensatory responses are biologically credible, two-tailed testing may be preferable, as it provides flexibility to detect effects in either direction and reduces the risk of overlooking unexpected but clinically meaningful associations, though at the cost of reduced power compared to a one-tailed test.

### 3.5. Error Types, Power, and Permutation Testing in VBA/IBDM

This section introduces the statistical framework for voxel-based analysis in radiotherapy dose–toxicity studies, emphasizing the need for rigorous error control when thousands of voxel-wise tests are conducted. It begins with clear definitions of Type I and Type II errors, family-wise error rate (FWER), false discovery rate (FDR), and statistical power, situating per-voxel and family-wise inference in the clinical context. The methodology of permutation testing is then developed, including variants of test statistics such as maxT, cluster-based inference, Threshold-Free Cluster Enhancement (TFCE), and Random Field Theory (RFT), while accounting for spatial dependence and recent adaptive approaches. Safeguards for enforcing directionality in biological hypotheses are discussed, followed by approximate power calculations and worked examples, the impact of dose grid size on sensitivity and specificity, and practical critiques and implications for reproducibility.

Error definition: Errors in hypothesis testing are classically divided into two categories. A Type I error, denoted α or a false positive, occurs when the null hypothesis is rejected even though it is true. In radiotherapy this would mean falsely concluding that a voxel or region is dose–toxicity-related, which can mislead clinical interpretation and treatment planning. A Type II error, denoted β or a false negative, occurs when the null hypothesis is not rejected despite a true effect being present. This represents a missed true association, which reduces sensitivity but generally has fewer safety consequences than false positives.

Error rates in multiple testing: Because voxel-based analysis involves thousands of simultaneous tests, false positives can accumulate rapidly unless carefully controlled. The family-wise error rate (FWER) quantifies the probability of at least one false positive across the entire image, given by FWER = 1 – P (no false positives) = 1 − (1 − α)^m^ where m is the number of tests. For example, in a 90 × 90 grid containing 8100 voxels, if α = 0.05 per test, the probability of observing zero false positives is (1 − 0.05)^8100^ ≈ 3.6 × 10^−181^, essentially zero, so the FWER approaches 100%. To reduce this risk, multiple testing corrections such as Bonferroni or permutation-based procedures are applied. The false discovery rate (FDR) takes a different perspective, controlling the expected proportion of false positives among voxels declared significant. For example, if FDR = 0.05, then on average 5% of the declared discoveries are expected to be false. FDR thus improves sensitivity compared with FWER but allows some false positives.

Power definition: Statistical power, defined as 1 − β, quantifies the probability of correctly detecting a true effect. In the context of VBA, per-voxel power refers to the probability of detecting a true effect at a specific voxel and measures sensitivity at individual sites, addressing the question of the likelihood of observing a significant result at that voxel if a true effect exists. In contrast, family-wise power denotes the probability of detecting at least one true effect anywhere in the image or dose distribution, reflecting the probability of identifying at least one significant voxel across the analysis when true effects are present. Family-wise testing requires accounting for thousands of simultaneous voxel-level comparisons, resulting in a stricter significance threshold. Consequently, family-wise power is generally lower than per-voxel power. In this context, “lower” indicates that the probability of detecting a true effect is reduced when transitioning from per-voxel to family-wise assessment. For example, per-voxel power may be 90% for a particular voxel, meaning there is a 90% chance of detecting a true effect at that location, whereas family-wise power, which accounts for multiple comparisons across the entire image, may decrease to 70%; even when true effects exist in some voxels, the stricter threshold reduces the likelihood of detecting any voxel. Thus, more stringent criteria reduce the probability of detecting true effects compared with examining individual voxels. This distinction is analogous to evaluating whether a dose–toxicity signal can be detected in one predefined region of the bladder (per-voxel power) versus determining whether any region of the bladder shows a signal when the entire organ is scanned while controlling for false positives (family-wise power). The latter approach is more demanding because the detection threshold is stricter, but it yields a more reliable assessment of whether clinically meaningful effects are present at the patient level.

How error control strategies influence power: Error control strategies directly influence these measures. Strict FWER control reduces both per-voxel and family-wise power by flagging only the strongest effects, while false discovery rate (FDR) control increases sensitivity but accepts some false positives. The choice of method therefore balances rigor against sensitivity, depending on study goals.

How family-wise power is practically estimated: Family-wise detection power is typically established through simulation. A known effect size (e.g., Δ = 2 Gy or 4 Gy) is injected into a region of interest, and the full permutation pipeline is rerun to evaluate how often the injected effect is detected. Repeating this process 1000 or more times with varied, realistic injection patterns yields stable estimates. Family-wise power is then defined as the proportion of simulations in which the injected effect is successfully identified, providing a practical measure of the ability to detect clinically meaningful dose differences in patients who develop toxicity. Per-voxel power is discussed in Section 3.5.5.

#### 3.5.1. Permutation Testing

Permutation testing has become central in VBA/IBDM because it calibrates inference to the empirical null distribution rather than assuming independence. Labels are randomly permuted while preserving the spatial correlation of voxel values, and the maximum statistic from each permutation is stored. This generates a maximum-statistic null distribution that provides valid FWER control. Compared with Bonferroni correction, which simply divides α by the number of voxels, permutation testing better preserves power by accounting for spatial correlations. For example, in 1000 voxel-wise tests under the null, Bonferroni correction reduces the per-test threshold to 0.05/1000 = 5 × 10^−5^, practically eliminating detections, whereas the Benjamini–Hochberg procedure at FDR = 0.05 might allow around 200 detections with roughly 10 false positives. Permutation testing lies between these extremes, more conservative than BH but far less severe than Bonferroni and, importantly, valid even under voxel dependence [38,39,110,111]. The number of permutations determines the smallest attainable *p*-value, approximately 1/(Npermutations + 1). Chen et al. [33] recommend around 1000 permutations, which yields a minimum *p* ≈ 0.001, while Puri et al. [45] examined as few as 10 and as many as 10,000 permutations and found that high-risk voxels identified on dose–toxicity maps were largely stable across these settings, although very small numbers of permutations produce unstable thresholds and coarse *p*-values.

#### 3.5.2. Variants of the Test Statistic

Several refinements to the standard max-T statistic have been developed. Chen et al. [33] implemented a permutation test comparing voxel-wise dose distributions between event and non-event groups, where each permutation produced average dose maps and their difference. Because raw voxel differences vary in variability, they standardized the differences by dividing by the permutation-wise standard deviation, then summarized with the maximum value to ensure Type I error control. Stepdown maxT [35] improves power by testing hypotheses sequentially and allowing less conservative thresholds as hypotheses are rejected. Cluster-based permutation tests [38] exploit spatial coherence by testing the size or mass of contiguous clusters rather than isolated voxels. Threshold-Free Cluster Enhancement (TFCE) [112] extends this idea by integrating across all thresholds, enhancing sensitivity when effects are diffuse. Random Field Theory (RFT) [113] provides a parametric framework but relies on smoothness assumptions and extensive pre-smoothing, which may not align with sharp gradients in radiotherapy dose maps.

#### 3.5.3. Spatial Dependence and Adaptive Methods

Dose maps exhibit strong spatial dependence due to beam geometry, the physics of dose deposition, and anatomical constraints. True dose–toxicity signals are thus more likely to appear as clusters rather than isolated voxels. Standard permutation-based approaches such as maxT implicitly account for dependence, but may be overly conservative when signals span contiguous areas. Methods that explicitly incorporate spatial structure, such as cluster-based tests or TFCE, improve sensitivity while maintaining rigorous error control and avoid the inflated false-positive rates observed in some parametric clustering approaches. Adaptive strategies have also been proposed. Sequential resampling methods terminate permutation once thresholds stabilize [114], improving efficiency. Structure-Adaptive Benjamini–Hochberg (SABHA) [115] and hierarchical FDR methods incorporate anatomical or spatial groupings to balance sensitivity and control. These developments show promise for imaging applications but require careful implementation to ensure validity.

#### 3.5.4. Enforcing Directionality

Many biological hypotheses in radiotherapy are directional, for example that higher dose increases toxicity. In one-tailed permutation testing, only maxima consistent with the hypothesis are recorded and thresholds are drawn from this distribution, improving power. In two-tailed tests, by contrast, the maximum absolute statistic is used, ensuring symmetric thresholds for both directions. Safeguards are needed to avoid spurious detections in the opposite direction; one option is to conduct complementary one-tailed tests in both directions and exclude contradictory results. One-tailed testing is more powerful under strict unidirectional hypotheses but may be misleading if effects are bidirectional or non-monotonic. In such cases, two-tailed inference provides a more cautious and transparent approach.

#### 3.5.5. Power Calculations and Critical Perspectives

Per-voxel power in permutation-based testing can be approximated analytically. As discussed in Section 3.4 and Section 3.5.4, the choice between one-tailed and two-tailed testing affects the permutation threshold t*, with one-tailed tests providing greater power when the directional hypothesis is correct. For a voxel with true mean difference Δ (in Gy) between event and non-event groups, the standard error of the group mean difference under Welch’s *t*-test is SE = √((s_1_^2^/n_1_) + (s_2_^2^/n_2_)), where s_1_ and s_2_ are between-subject standard deviations and n_1_ and n_2_ are the corresponding sample sizes. The signal-to-noise ratio is δ = Δ/SE. Empirically, 80% power is achieved when δ ≈ t* + 0.84, where t* is the permutation-derived threshold and 0.84 is the 80th percentile of the standard normal distribution. To achieve at least 80% power, the signal-to-noise ratio must satisfy δ ≥ t* + 0.84, which can be rearranged as Δ/SE ≥ t* + 0.84. Multiplying both sides by SE and then dividing by (t* + 0.84) yields Δ/(t* + 0.84) ≥ SE, or equivalently, SE ≤ Δ/(t* + 0.84). This means the standard error must not exceed the upper bound Δ/(t* + 0.84) to maintain adequate power. If SE is larger than this bound, the test is underpowered.

For illustration, in our previous study [18,45] analyzing urinary tract obstruction toxicity in a high-dose bladder region, the between-subject standard deviations were s_1_ = 5 Gy for events and s_2_ = 4 Gy for non-events, with n_2_ = 701. Based on 1000 permutations, the observed permutation distribution of maxT produced a maximum value of approximately 4.15. The threshold for significance, t*, was set at the 95th percentile of this null distribution, yielding t* ≈ 3.95 for a two-sided test. For a one-sided test, because only extreme values in the hypothesized direction are considered, the threshold derived from the corresponding one-sided permutation distribution was t* ≈ 3.70. The standard error expression is therefore SE = √(25/n_1_ + 16/701). For a two-sided analysis with t* = 3.95, the relevant threshold is t* + 0.84 = 4.79; with Δ = 4 Gy this gives a bound of SE < 0.835 Gy, which requires n_1_ ≥ 38, and with Δ = 2 Gy the bound is SE < 0.418 Gy, which requires n_1_ ≥ 166. For a one-sided analysis with t* = 3.70, the relevant threshold is t* + 0.84 = 4.54; with Δ = 4 Gy the bound is SE < 0.881 Gy, requiring n_1_ ≥ 34; and with Δ = 2 Gy the bound is SE < 0.441 Gy, requiring n_1_ ≥ 146. Thus, with approximately 62 events and 701 non-events, the study was well powered (>80%) to detect voxel-level differences of 4 Gy but underpowered to detect differences of 2 Gy. A summary for other sample sizes is reported in Table 1. This analytic approach estimates power at the per-voxel level. By contrast, family-wise detection power (the probability of detecting at least one true effect anywhere in the image) typically requires simulation-based evaluation, as described in Section 3.5.

Dose grid size also influences statistical inference. Voxel size refers to the sampling density of the calculated dose grid, typically 2–3 mm isotropic, and should not be conflated with the physical resolution of dose delivery, which depends on beam-shaping hardware such as multileaf collimator (MLC) leaf width or proton spot size [116,117,118,119]. The commonly used term “voxel resolution” can be misleading: decreasing voxel size in the dose grid does not improve the true physical resolution of the delivered dose, it only samples the same underlying distribution more finely. Statistically, smaller voxels increase the number of tests, lengthening null distribution tails and making thresholds more conservative, improving control of false positives but reducing sensitivity to smaller clusters of dose–toxicity effects. Larger voxels reduce the number of tests, improving sensitivity but sacrificing spatial specificity by averaging across larger regions. For example, a 240 × 240 × 240 mm^3^ volume contains roughly (240/3)^3^ = 512,000 voxels at 3 mm resolution but only (240/6)^3^ = 64,000 voxels at 6 mm, an eightfold reduction. This substantially reduces the multiple comparisons burden, easing significance thresholds but blurring spatial detail. Thus, voxel size in dose–toxicity analysis must be chosen with attention both to the physical limits of dose deliverability and to the biological interpretability of the results.

Critiques of permutation-based methods highlight that while strict FWER control protects against false positives, it may obscure moderate but clinically important associations, reducing both per-voxel and family-wise power. Cluster- and region-based approaches such as TFCE improve sensitivity by pooling information across voxels, but parametric clustering risks inflated false positives and extended signals may be biologically ambiguous. Reproducibility depends on transparent reporting and sensitivity analyses. Ultimately, error control strategies in VBA must balance minimizing Type I errors with maintaining adequate power, considering spatial dependence, effect size, voxel sampling, and clinical interpretability.

### 3.6. Using Dose-Only Data as the Predictor of Post-Radiotherapy Toxicity

Relying on spatial dose alone to predict toxicity is a major limitation of many current VBA/IBDM studies. Toxicity is a multifactorial outcome shaped by patient biology, baseline function, anatomy, treatment technique, and care pathways. Ignoring these factors can lead to biased or unstable dose–toxicity associations, which may differ across cohorts and, in some cases, appear biologically implausible.

Baseline confounding is a key issue. For example, in prostate cancer, genitourinary toxicity risk depends strongly on pre-existing urinary symptoms, prior interventions such as transurethral resection of the prostate (TURP), and dose hotspots [19,120,121,122]. Without adjusting for these factors, pre-existing dysfunction may be misattributed to radiation, inflating associations in predisposed regions [19,123]. Baseline correction (e.g., subtracting pre-treatment grade from post-treatment grade) helps by focusing on radiation-related change, but it comes with important caveats. By reducing the number of apparent events, it decreases statistical power and can shrink significant regions. More critically, if radiation-induced toxicity builds upon baseline dysfunction, correction may remove causally relevant information, discarding part of the signal of interest. In such cases, baseline adjustment can paradoxically obscure genuine dose–toxicity relationships. A balanced strategy is therefore needed: analyses should report both absolute post-treatment outcomes and baseline-adjusted change, and sensitivity analyses should explore whether results are robust to the choice of correction method, including examination of significant clusters and their adjacent regions. This dual reporting avoids attributing baseline morbidity to radiation while guarding against erasure of clinically relevant additive effects.

Genetic heterogeneity further complicates interpretation of radiosensitivity, which varies between patients due to inherited differences in DNA repair, inflammatory signaling, and other pathways [84]. Some individuals develop toxicity at relatively low doses, while others tolerate higher doses without harm. Polygenic risk score (PRS) aggregate small effects from many variants into an overall measure of inherited susceptibility. In prostate cancer, incorporating PRS into dose-only spatial models eliminated high-risk calls in low-dose regions and localized predicted high-risk regions to high-dose areas, consistent with the expectation that toxicity increases with dose [92,93,124]. This suggests that low-dose associations observed in dose-only models were artifacts of unmeasured genetic radiosensitivity rather than true low-dose effects. However, PRS do not capture many other influences, including clinical, demographic, and environmental factors [125,126,127,128,129]. As datasets grow, multifactorial risk score (MRS) that integrate genetic markers, clinical variables, and dose are likely to be more informative than models based on PRS, MRS alone, or dose alone. For example, one study [130] combined a PRS derived from 38 SNPs with clinical factors, including age, T stage, comorbidities, and lifestyle variables, and temporal lobe dosimetric parameters to predict radiation-induced brain injury. Integrating dose with genetic and clinical variables may further refine both the location and magnitude of predicted high-risk regions, revealing patterns that could be missed by single-factor models [131].

**Figure 8:**
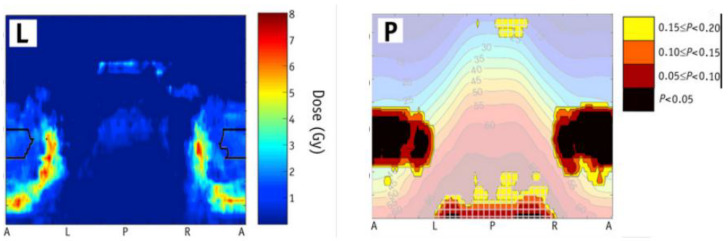
Pixel-wise dose maps compare patients with significant increases in the International Prostate Symptom Score (ΔIPSS10, classified as events) to those without such increases (non-events). The left panel (L) presents voxel-level dose difference maps (events minus non-events), highlighting regions with statistically significant differences using univariable Wilcoxon signed-rank tests. The right panel (P) shows areas identified using multivariable logistic regression or event-count models that adjust for non-dosimetric covariates (such as baseline symptoms, trial arm, and number of follow-up visits) but exclude genetic data. In this panel, regions with negative regression coefficients indicate that higher radiation doses were associated with lower toxicity risk. Reproduced from Yahya et al. [132] with permission from Elsevier.

Counterintuitive inverse associations can also appear when multivariable models are used without genomics. Yahya et al., [132] using models with clinical covariates but no genomic data, reported bladder regions where higher dose correlated with lower toxicity risk (Figure 8). Such patterns may arise when treatment plans deliberately reduce dose to anatomically sensitive areas, creating an apparent “protective” effect. Even in the absence of deliberate dose reductions, inverse associations can emerge due to unmeasured or unaccounted-for covariates that could jointly influence both dose distribution and post-radiotherapy toxicities [133,134].

VBA/IBDM is valuable because it localizes exactly where these unexpected associations occur, enabling targeted sensitivity analyses. For example, investigators can re-run models excluding patients with atypical anatomy, stratify by bladder volume, or test robustness after adjusting for additional covariates. If the inverse association disappears under these perturbations, it is more likely an artefact than a true protective effect. Without such checks, findings of “higher dose associated with lower toxicity risk,” particularly in low- or moderate-dose regions, should be interpreted with caution.

These examples illustrate a broader point: even statistically sophisticated multivariable methods can produce flawed results if model structure is poorly matched to the data or key factors are excluded. Yule–Simpson’s paradox is a classic risk: an association observed at the group level can reverse or disappear after stratifying by a confounder. In dose–toxicity modeling, adjusting for baseline symptoms, anatomy, or genetic susceptibility may change the sign or magnitude of dose effects [46,135,136,137,138].

In summary, dose provides essential but incomplete information about toxicity risk. Dose-only models can reveal spatial differences, but without accounting for factors such as baseline, clinical or genomics, they are prone to confounding and misinterpretation. Incorporating appropriate covariates, modeling nonlinearities and interactions, reporting absolute dose alongside statistical results, and testing on independent data are necessary to produce clinically credible, biologically meaningful maps.

### 3.7. Safeguards in Dose–Toxicity Modeling

To ensure robust and biologically credible voxel-wise inference, several methodological and reporting safeguards should be applied. The primary safeguard is incorporating absolute dose values directly, because biological responses depend fundamentally on absolute radiation exposure, whereas t- and U-based tests are scale-invariant. Reporting absolute dose ranges for significant voxels (e.g., mean/median and standard deviation/interquartile range in Gy) helps contextualize statistical findings; a significant difference at 2 Gy may be clinically irrelevant for late bladder toxicity, whereas differences around 10 Gy are more meaningful. Significant voxels should be overlaid on dose maps, but only those receiving biologically meaningful doses should be highlighted, so that statistically significant voxels with negligible exposure are not misinterpreted as clinically relevant.

When using equivalent dose models such as EQD2, robustness to different α/β assumptions should be evaluated across a plausible range (e.g., 1–5 Gy for late-responding tissue and 6–10 Gy for acute-responding tissue). Stability of significant voxel patterns across this range increases confidence in results, while marked shifts in location, extent, or magnitude indicate biological uncertainty. Minor shifts, such as small changes in voxel counts or cluster boundaries that do not alter the main interpretation, can be considered stable, whereas major changes (e.g., cluster disappearance or >20–30% change in voxel count) suggest instability. Quantitative measures such as the Dice similarity coefficient can aid in distinguishing stable versus unstable regions [45]. Additionally, sign consistency within clusters should be verified: positive test statistics indicate higher dose associated with increased toxicity risk, negative statistics suggest the opposite, and clusters containing mixed directions are more likely to reflect noise or artifacts than true effects. Detected regions should align with anatomically plausible high-dose areas consistent with treatment geometry.

For cross-cohort comparisons, it is essential to assess common support in EQD2 distributions. Voxel-wise comparisons are meaningful only if the dose ranges substantially overlap between cohorts; otherwise, observed differences may reflect variations in treatment protocols, prescription, or delivery rather than genuine biological differences. This consideration applies both to cohorts treated at different centers with differing dose regimens and to cohorts within the same center receiving distinct treatment plans. When overlap is limited, trimming to the common EQD2 range and explicitly modeling cohort effects (e.g., stratification or cohort-specific slopes) helps prevent artefactual findings. Reporting the extent of overlap, the dose range used for inference, and whether results remain stable after trimming ensures transparency and enhances biological plausibility. Equality claims across cohorts are inappropriate unless dose distributions have been harmonized.

Methodologically, robustness can be further improved by incorporating relevant covariates known or suspected to influence toxicity, including baseline symptom scores, tumor characteristics (e.g., size, stage, or grade), prior procedures (e.g., TURP), acute treatment effects, age, comorbidities, medications, and anatomical descriptors (e.g., bladder volume or filling pattern), as well as genomic variables such as PRS. Tumor characteristics are particularly important because they influence both treatment planning and dose distribution, which in turn affect tissue toxicity. Where feasible, combining dose with broader MRS frameworks that integrate genetic and non-genetic predictors provides a more comprehensive assessment of individual susceptibility.

Flexible dose modeling may capture nonlinear effects and interactions with patient factors (e.g., dose × baseline symptom). Logistic regression or other generalized linear models can be applied at the voxel level, including continuous dose, relevant covariates, and pre-specified interactions, with penalization methods used to limit overfitting when analyzing large numbers of voxels. Sensitivity analyses should be undertaken whenever possible, such as repeating models with and without baseline correction, varying α/β for EQD2, excluding voxels below biologically plausible dose floors, comparing parametric and non-parametric tests, or evaluating robustness to registration and dose–surface map methods [36].

Finally, findings should be evaluated through internal cross-comparison within the development dataset and subsequently tested in independent external cohorts. While ‘external validation’ is more accurately described as ‘external comparison’ (since concordance across datasets demonstrates reproducibility rather than clinical validity) the principle is to determine whether results generalize beyond the original cohort. Ultimately, prospective plan adaptation studies with clinical outcome evaluation are required to establish whether sparing VBA/IBDM-identified regions actually reduces toxicity, thereby demonstrating the clinical value of voxel-wise dose–toxicity associations.

### 3.8. Association Does Not Imply Causality

VBA/IBDM generate statistical maps that highlight regions where local dose associated with toxicity. These maps are valuable for hypothesis generation but do not necessarily establish causation. A statistical association may arise for reasons unrelated to irradiation. For example, voxels near tumors often receive higher doses due to planning constraints and may also belong to tissues intrinsically vulnerable or impaired. Apparent patterns may reflect consistent planning practices rather than regional susceptibility.

Spatial interference further complicates interpretation: the effect of dose at one voxel may depend on doses to neighboring voxels, yet voxel-wise analyses typically ignore such dependencies. Other mechanisms can create or distort associations. Dose distribution and toxicity risk may be jointly influenced by external factors, creating spurious associations. Collider bias can arise when adjusting for variables affected by both dose and toxicity; for instance, follow-up imaging may be more frequent in patients receiving higher doses or those at elevated risk due to tumor characteristics, and conditioning on such variables can generate artificial associations. Measurement errors in dose delivery pathway may smear true signals or produce false clusters. Multiple testing across thousands of voxels can yield false positives even after statistical correction. Temporal dynamics of toxicity, arising from acute effects, tissue healing, and adaptive responses, further complicate causal interpretation. Adjusting for mediators or downstream colliders can inadvertently block causal pathways or introduce new biases; only pre-exposure confounders should generally be adjusted.

Careful framing is critical. Even prospective dose-sparing trials cannot prove that specific regions cause toxicity, though they can evaluate clinical utility: whether sparing these regions improves outcomes. Distinguishing causality from utility prevents over-interpretation while supporting pragmatic clinical evaluation.

Explicitly defining the causal question, or estimand (i.e., precisely what effect is being estimated), is essential. For example, one might ask: how does the probability of rectal bleeding change if dose to a specific subregion were reduced by 5 Gy, holding other factors constant? Without a clear estimand, results remain ambiguous. Observational data often violate key assumptions for causal inference. A key challenge for causal inference is the assumption of positivity, which requires that all relevant dose levels occur in the data for the voxel or region of interest. In practice, some regions (such as voxels adjacent to tumors) almost always receive high doses. Because few or no patients receive lower doses in these regions, it is impossible to observe what would have happened under a lower dose (the counterfactual outcome). Without this variation, causal effects cannot be reliably estimated from observational data.

A variety of causal inference methods such as directed acyclic graphs (DAGs), g-methods, instrumental variables, natural experiments, and regression discontinuity designs [139,140,141,142,143,144,145,146,147], which can support interpretation. While these approaches cannot transform VBA/IBDM into causal analyses, they help assess whether observed associations are plausible once confounding and bias are addressed. DAGs, in particular, clarify assumptions and distinguish pre-exposure confounders (which should be adjusted for) from mediators or colliders (which should not). For instance, tumor size may confound the relationship between rectal dose and bleeding, whereas acute rectal toxicity lies on the causal pathway and should not be adjusted for.

## 4. Why Is Dose–Survival Modeling Not Discussed?

This perspective deliberately focuses on dose–toxicity modeling rather than dose–survival to address methodological challenges in a more controlled and tractable setting. Overall survival is shaped by a complex interplay of tumor control, systemic therapies, patient-specific responses to subsequent treatments, non-cancer mortality, and treatment selection biases. The pathway from radiation dose to survival is therefore indirect, mediated through many factors beyond local radiation injury, and requires distinct analytical frameworks that address competing risks, time-varying confounders, and probabilistic outcomes, considerations beyond the scope of this work.

Although several studies have reported associations between cardiac substructure dose and survival [41,148,149], these were designed around dose–survival rather than dose–toxicity questions. Patients were stratified by survival rather than by evidence of radiation-induced injury, so regions of high dose may reflect broader prognostic factors, such as disease stage or systemic health rather than radiation-induced toxicity. In some studies, univariable voxel-wise analyses were facilitated by unusually large intergroup dose differences (e.g., median differences up to 18.5 Gy between survival groups). Such stark contrasts are uncommon for most organs at risk, where spatial dose variation is more subtle and biological effects are diffuse, limiting reproducibility.

Extending spatial modeling to survival introduces substantial additional complexity. Non-proportional hazards can arise due to time-dependent treatment effects, and competing risks complicate interpretation when death may result from multiple causes. Immortal time bias may occur when survival is guaranteed during exposure periods, and time-varying confounders can further distort associations. Moreover, statistical power requirements (often ≥200 patients with ≥20% events) become challenging to meet, necessitating larger cohorts and longer follow-up to observe sufficient events. The multifactorial nature of survival, influenced by disease stage, systemic therapies, and comorbidities, increases both confounding and validation requirements, raising the risk that findings are misinterpreted as causal rather than reflecting broader prognostic factors.

By focusing on dose–toxicity rather than survival, this perspective prioritizes resolving fundamental methodological challenges in a more tractable context. Future work should aim to integrate robust dose–toxicity models with survival outcomes once methodological challenges such as reproducibility, confounding control, and statistical inference are adequately addressed.

## 5. Limitations

This work has several limitations. It is not a systematic survey of all VBA/IBDM studies, and the focus on seven methodological challenges reflects the authors’ experience and identified priorities. Other important issues and alternative frameworks may yield different insights. Emerging methods from machine learning, deep learning, and advanced statistical approaches for high-dimensional spatial data are not covered, though they may help address some limitations [150,151,152,153,154,155,156,157,158,159,160]. Because many challenges are interdependent, a definitive sequence or prioritization for addressing them cannot be provided. While some guidance is offered to help avoid basic technical pitfalls, a technically standardized roadmap for implementation has yet to be developed.

The cost-effectiveness of VBA/IBDM compared with simpler DVH-based methods remains insufficiently studied, raising the question of whether the added complexity provides proportional clinical benefit. Costs associated with large patient cohorts, extended follow-up, genomic profiling, advanced imaging, or computing infrastructure are not evaluated. Fundamental preprocessing issues, such as registration accuracy, scanner-dependent noise, partial-volume effects, interpolation artifacts, dose reconstruction uncertainties or motion or delivery artifacts [161], were acknowledged but not analyzed in depth, even though they can substantially influence voxel-wise results and risk creating spurious associations.

Although the need for clinical validation is highlighted, detailed study designs, outcome measures, interim milestones, success criteria, and structured validation strategies for transitioning from exploratory to directive clinical use are not provided. The methodological challenges discussed are conceptual and broadly applicable across disease sites and modalities, although practical implementation details may vary. Finally, the discussion treats dose–toxicity relationships as largely static, leaving the dynamic interplay of acute effects, healing, late toxicities, timing of outcome assessment and dose-rate effects insufficiently addressed.

## 6. Conclusions

VBA/IBDM have opened new avenues for studying spatial dose–toxicity relationships in radiotherapy. When applied rigorously, these approaches can highlight subregions within OARs that may disproportionately contribute to treatment-related toxicity, thereby supporting the design of safer and more personalized dose distributions. The ultimate clinical ambition is to tailor radiotherapy on a patient-specific basis, balancing tumor control with minimized acute and late toxicity post-radiotherapy.

Despite the growing research interest, the current body of VBA/IBDM evidence remains constrained by methodological limitations and insufficient clinical validation or evidence of clinical value. Technical gaps such as ambiguity in selecting between parametric versus non-parametric frameworks, inconsistency in the use of one- versus two-tailed hypothesis testing, and inadequate attention to statistical invariance under uniform dose scaling, are not minor issues. Inappropriate statistical choices can yield paradoxical or uninterpretable results, undermining reproducibility and credibility. Without methodological standardization, it will remain difficult to attract funders and justify investment in the prospective clinical trials needed for clinical translation of this technique.

Equally concerning is the over-reliance on dose-only toxicity prediction models, which risks neglecting the multifactorial nature of post-radiotherapy toxicities. Future progress requires a decisive shift from simple, univariable, dose-only models toward integrative, multivariable dose–toxicity frameworks, as previously discussed outside the VBA/IBDM context [105,162,163,164]. Univariate, dose-only models should primarily serve as hypothesis-generating tools to identify candidate spatial relationships and guide future research directions. In contrast, multivariate models that integrate dose with genomic features represent the pathway toward clinical translation, though only after rigorous validation and/or demonstrated clinical benefit in prospective studies. In particular, the incorporation of genomic features, such as PRS or MRS, represents a critical next step. Such integration would enable models to account for inter-individual radiosensitivity and biological predisposition to toxicity. When combined with clinical covariates (e.g., baseline toxicity, comorbidities, concurrent therapies, and anatomical variability), these multivariable models would substantially enhance both the biological plausibility and predictive performance of dose–toxicity mapping.

Equally important is the adoption of causal inference-based analytic strategies, which move beyond correlative associations to disentangle true dose–toxicity mechanisms from confounding or spurious effects. By embedding VBA/IBDM within a causal framework, findings can become more robust, clinically interpretable, and ultimately actionable. To ensure reliability and comparability across studies, harmonized protocols for data processing (such as image registration, voxel size standardization, dose–surface mapping, statistical analysis, interpolation, or multiple-comparison correction) must be implemented consistently to enable external comparison in independent patient cohorts and validation to establish clinical value.

For future studies, including an expert medical statistician as a critical project team member who may help minimize methodological errors and ensure appropriately rigorous study designs and analyses. Researchers should provide comprehensive details on data inclusion and exclusion criteria for VBA/IBDM analyses, along with the parameters chosen for data processing, to enhance reproducibility and enable meaningful cross-study comparisons. To avoid potential pitfalls associated with un-validated in-house toolkits, research groups are encouraged to leverage established open-source libraries and frameworks that have undergone extensive community testing and peer review. Within the Python ecosystem, comprehensive tools are available for VBA/IBDM analyses, including core numerical computing libraries (NumPy and SciPy), data handling packages (pandas), visualization tools (matplotlib, seaborn, and plotly), statistical analysis libraries (statsmodels, pingouin, and scikit-learn), medical image processing packages (SimpleITK, ITK, and Nibabel), radiotherapy-specific libraries (pydicom, dicompyler-core, rt-utils, pymedphys, and pyradiomics), neuroimaging statistical packages with permutation testing capabilities (MNE and nilearn), performance optimization tools (joblib and numba, dask), interactive visualization packages (napari), and testing frameworks (pytest and hypothesis). When specialized requirements necessitate custom development, existing resources should be extended or adapted whenever possible. If in-house tools are ultimately developed, their performance must be benchmarked against other open-source alternatives using similar methodologies (e.g., rtdsm [165] or VoxelStats [166] or R-based tools for dose-response analysis [167,168]) with simulated or well-characterized datasets, and the software tools, detailed methodologies implemented, and benchmarking results should be published to ensure transparency and enable reproducibility of the findings. The use of closed-source tools, such as WordlMatch [169] or Manchester Tool Kit [170], should be kept to a minimum.

It is important to recognize that, despite current limitations, VBA/IBDM approaches have already yielded valuable hypothesis-generating insights. This perspective has outlined key methodological challenges while emphasizing that these techniques hold substantial promise as tools for precision radiotherapy. At present, however, their role should remain exploratory: results are often overstated without adequate validation or demonstration of clinical utility, underscoring the need for caution. Moving forward, progress requires a two-tiered strategy. First, rigorous technical standardization, including harmonized analysis pipelines, must be implemented to ensure reproducibility and credibility. Second, research should advance toward integrative, multivariable, and causal modeling frameworks, supported by broad external comparison across independent cohorts. Only through coordinated achievement of both tiers can VBA/IBDM evolve from exploratory instruments into clinically actionable tools, capable of safely and effectively informing routine treatment decisions in radiotherapy.

## Figures and Tables

**Table 1:** This table summarizes the minimum number of events (n_1_) required to achieve approximately 80% per-voxel power at α = 0.05 using the permutation max-T procedure. For a two-sided test with t* = 3.95, the maximum allowable standard error is SE = 0.835 Gy for Δ = 4 Gy and SE = 0.418 Gy for Δ = 2 Gy. For a one-sided test with t* = 3.70, the maximum allowable standard error is SE = 0.881 Gy for Δ = 4 Gy and SE = 0.441 Gy for Δ = 2 Gy. Power is approximated by the relation δ ≈ t* + 0.84, where 0.84 is the 80th percentile of the standard normal distribution (Z = 0.84), and the signal-to-noise ratio is defined as δ = Δ/SE, with Δ representing the true per-voxel mean difference between groups (Gy) and SE given by Welch’s formula SE = √(s_1_^2^/n_1_ + s_2_^2^/n_2_). Note that the n_1_ values in the table depend on n_2_ as well as the assumed between-subject standard deviations (s_1_ = 5 Gy for events, s_2_ = 4 Gy for non-events). SE = standard error; Δ = effect size (dose difference in Gy); t* = permutation-derived critical statistic controlling multiple comparisons; n_1_ = number of events; n_2_ = number of non-events.

One-Sided n_1_ (Δ = 2 Gy)	One-Sided n_1_ (Δ = 4 Gy)	Two-Sided n_1_ (Δ = 2 Gy)	Two-Sided n_1_ (Δ = 4 Gy)	n_2_ (Assume n_2_ Fixed for the Calculation of n_1_)
734	41	1744	47	100
220	36	266	41	200
178	35	207	39	300
163	34	187	39	400
155	34	176	38	500
150	34	170	38	600
147	34	166	38	700

## Data Availability

Not applicable.
